# Clustering of Multiple Psychiatric Disorders Using Functional Connectivity in the Data-Driven Brain Subnetwork

**DOI:** 10.3389/fpsyt.2021.683280

**Published:** 2021-08-18

**Authors:** Tomoki Tokuda, Okito Yamashita, Yuki Sakai, Junichiro Yoshimoto

**Affiliations:** ^1^Brain Information Communication Research Laboratory Group, Advanced Telecommunications Research Institute International, Kyoto, Japan; ^2^Okinawa Institute of Science and Technology Graduate University, Okinawa, Japan; ^3^Center for Advanced Intelligence Project, RIKEN, Tokyo, Japan; ^4^Division of Information Science, Graduate School of Science and Technology, Nara Institute of Science and Technology, Nara, Japan

**Keywords:** clustering, functional connectivity, biomarker, multiple clustering, psychiatric disorder

## Abstract

Recently, the dimensional approach has attracted much attention, bringing a paradigm shift to a continuum of understanding of different psychiatric disorders. In line with this new paradigm, we examined whether there was common functional connectivity related to various psychiatric disorders in an unsupervised manner without explicitly using diagnostic label information. To this end, we uniquely applied a newly developed network-based multiple clustering method to resting-state functional connectivity data, which allowed us to identify pairs of relevant brain subnetworks and subject cluster solutions accordingly. Thus, we identified four subject clusters, which were characterized as major depressive disorder (MDD), young healthy control (young HC), schizophrenia (SCZ)/bipolar disorder (BD), and autism spectrum disorder (ASD), respectively, with the relevant brain subnetwork represented by the cerebellum-thalamus-pallidum-temporal circuit. The clustering results were validated using independent datasets. This study is the first cross-disorder analysis in the framework of unsupervised learning of functional connectivity based on a data-driven brain subnetwork.

## 1. Introduction

Abnormal functional connectivity (FC) in the brain has been extensively studied for a better understanding of psychiatric disorders (1–3). Typically, an FC study focuses on a particular psychiatric disorder, and reports the brain regions related to abnormal FC for psychiatric disorders. The results of these individual studies are not necessarily consistent, even for the same psychiatric disorder (4, 5). Nonetheless, several meta-analyses imply that there may be shared brain regions of abnormal FC that are related to different psychiatric disorders. A meta-analysis focusing on the default mode network (DMN) (6) suggests that the DMN is a consistent biological correlate of various psychiatric disorders, including major depressive disorder (MDD), bipolar disorder (BD), and schizophrenia (SCZ). Furthermore, a meta-analysis focusing on psychomotor systems, including the DMN (7), suggests that the balance in psychomotor mechanisms may determine MDD, BD, and SCZ. Recently, a large sample study by (8) showed that shared connectomic abnormalities among MDD, BD, and SCZ are bilateral thalamus, cerebellum, frontal pole, supramarginal gyrus, postcentral gyrus, lingual gyrus, lateral occipital cortex, and parahippocampus. Another recent large sample study by (9) showed that the common abnormality among MDD, BD, and SCZ is frontoparietal network connectivity. In contrast, in non-FC based studies, a genome-wide association study (10) showed substantial overlap of genetic influences among MDD, BD, and SCZ. A meta-analysis by (11) showed that gray matter density decreased in the dorsal anterior cingulate and right/left insula for MDD, BD, SCZ, addiction, obsessive-compulsive disorder, and anxiety disorders. In a large sample study (12), it was shown that SCZ, BD, and ASD subjects shared similar white matter microstructural differences in the body of their corpus callosum, as compared to healthy subjects. Such cross-disorder analysis is vital for a comprehensive understanding of various psychiatric disorders and for deepening our understanding of a particular psychiatric disorder. In the present study, we aimed to perform cross-disorder analysis using a novel unsupervised approach to reveal the underlying shared functional connectivity related to psychiatric disorders.

Typically, to elucidate the relevant functional connectivity for a psychiatric disorder, FC is contrasted between patients and healthy control (HC) subjects using various machine learning techniques in a supervised manner (13–17). Diagnostic label information is used as the response variable in supervised learning, which is based on clinical criteria such as the Diagnostic and Statistical Manual of Mental Disease (DSM) (18). DSM diagnosis defines various types of psychiatric disorders based on several clinical symptoms that are shared by these psychiatric disorders. It relies on clinical interviews to which patients respond, which makes the diagnosis subjective by nature. Moreover, various psychiatric disorders share common cognitive deficits with high comorbidity across psychiatric labels, which raises questions about the underlying structure and assumptions of the classification (19, 20). All these aspects of DSM diagnosis imply that the diagnostic label does not necessarily denote the “ground truth” (21).

To overcome this problem of the diagnostic label, it will be of interest to perform unsupervised analysis, that is, cluster analysis. Combined with the feature selection procedure, the unsupervised method allows the identification of functional connectivity related to psychiatric disorders, without explicitly using psychiatric labels. Such an approach is in line with the dimensional approach proposed by the Research Domain Criteria (RDoC), which is based on the mechanism of disorders rather than their symptoms (22). Moreover, it is quite useful to perform a cluster analysis that includes multiple psychiatric disorders because it enables us to reveal a common or different functional connectivity for cross-disorder analysis without directly using psychiatric labels. Nonetheless, cluster analysis for cross-disorders is currently limited to clinical data, such as symptom data, genetic data, and EEG data only (23–25). Though several studies have performed FC-based cluster analysis for a single disorder (26, 27), to the best of our knowledge, no study for cross-disorders has performed cluster analysis using FC data.

The objective of the present study was hence to examine whether there is a common functional connectivity related to various psychiatric disorders. We performed a cross-disorder analysis using FC data in an unsupervised manner. To this end, we applied the ROI-based multiple clustering method, which has been recently developed specifically for clustering functional connectivity matrices (28). This ROI-based multiple clustering method is unique because it optimally divides ROIs into several subsets; for each subset of ROIs, an optimal cluster solution is identified accordingly. In the present paper, we refer to each subset of ROIs as a “view” in which the terminology carries connotations that help us view only a particular set of ROIs for a single clustering. For multiple clustering, we identify multiple views in which subject clustering is performed separately. The ROI-based method that we use optimizes both view structures and subject clustering in each view simultaneously (for more details, please see section 2.1). This specific aspect of the method enables us to identify a data-driven brain subnetwork that is relevant to subject cluster patterns. Furthermore, this method reduces the search space of parameters from combinations of connectivity to combinations of nodes, enabling efficient inferences of clustering for high-dimensional FC data. We applied this method to the FC dataset consisting of 322 subjects with various psychiatric disorders. For a specific brain subnetwork, there were four clusters characterized by MDD, young HC, SCZ/BD, and ASD, respectively. To examine the reproducibility of the clustering results, we applied the yielded model of classification to independent data, which largely confirmed the reproducibility of the results.

In the following sections, we first outline the multiple clustering method, which is unique to the present study. Second, we describe the datasets for both discovery and validation. Third, we analyze the clustering results for discovery data and classification results for the validation data. Finally, we discuss the interpretations of the clustering results and methodological novelty of the present study.

## 2. Materials and Methods

### 2.1. ROI-Based Multiple Clustering Method

In this study, we applied a recently developed multiple clustering method (28) to perform cluster analysis. Multiple clustering is generally based on the assumption that multiple cluster solutions of objects (subjects) exist in a given dataset, and there are several approaches to revealing the underlying multiple-view structure in data [for comprehensive reviews, see (29, 30)]. In the present study, we focused on “subspace clustering,” in which cluster solutions were obtained for several subspaces (i.e., subsets) of features. It was not known in advance which subsets of features should be used for optimal cluster solutions; hence, the multiple clustering method entailed the optimization of both (exclusive) feature partitioning and cluster solutions. The advantage of such an approach was that we did not discard from the analysis any irrelevant features for a particular cluster solution, but utilized these features for another cluster solution, which widened the scope of possibilities to identify optimal cluster solutions.

**Figure 1:**
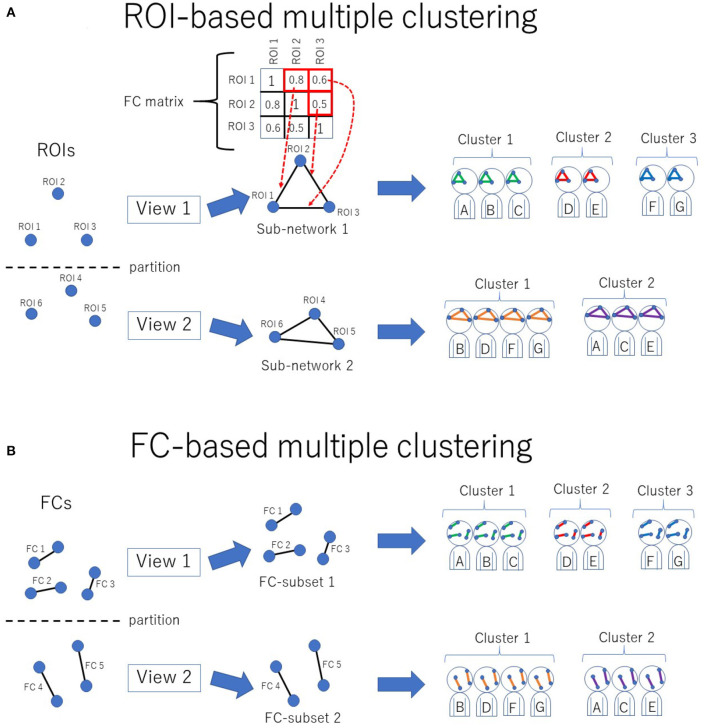
Conceptual illustration of multiple clustering methods. **(A)** ROI-based multiple clustering method. ROIs are partitioned into several groups (views). In each view, the subject cluster solution is obtained using a particular subset of ROIs (i.e., a subnetwork). The FC matrix represents connectivity within the subnetwork. For instance, in this illustration, the connectivity within view 1 consists of three ROIs, which are denoted by the 3 × 3 FC matrix. This method identifies optimal pairs of subnetworks and cluster solutions, where optimization is performed simultaneously for both the subnetwork and cluster solution. The color in the subnetwork for subjects A–G denotes a cluster-specific pattern of functional connectivity. For simplicity, there are two views in this example. **(B)** FC-based multiple clustering method. Instead of ROIs, FCs are exclusively partitioned for the subject cluster solutions. This method identifies optimal pairs of subsets of FCs and cluster solutions.

The novel multiple clustering method (28) was developed specifically for clustering subjects based on functional connectivity matrices without vectorization. The uniqueness of this method is its ROI-based approach rather than the conventional FC-based approach, which is achieved by fitting the data to the Wishart mixture model (hereafter referred to as “ROI-based multiple clustering method”). As an output, the method yields several pairs of relevant ROIs and subject cluster solutions (Figure 1A), where each pair is referred to as “view.” It is noteworthy that all FCs pertaining to a selected subset of ROIs are evaluated by fitting to the Wishart mixture model, which results in subnetwork identification. The number of views and clusters are automatically inferred in the nonparametric Bayesian framework (31), setting the concentration parameter for the Chinese restaurant process to one (32).

The key idea of this method is the assumption of independence between subnetworks, each of which consists of several ROIs. This assumption does not hold for real data because subnetworks in the brain are putatively interconnected in a complex manner (33). Hence, to meet this assumption, the “whitening” procedure is applied for the correlation matrices as a pre-processing requirement [for more details, please refer to (28)]. It is expected that the whitening procedure preserves cluster structures within subnetworks, whereas the functional connectivity between subnetworks becomes zero. Furthermore, it is expected that this procedure normalizes the correlation matrices such that it enhances the generalization of the yielded model.

One limitation of the method is that a conventional approach to removing the influence of confounding factors (e.g., age and sex) based on Generalized Linear Model (GLM) cannot be applied for pre-processing of FC. This is because the positive definiteness of the FC matrices would be lost by the application of the GLM. As an alternative approach, we consider the confounding factors in the *post-hoc* analysis (for more details, please refer to section 4.4).

The optimization strategy of the method was based on a greedy search, which was initialized with a random configuration of views and clusters. We set the number of initializations to 1,000, which in turn yielded 1,000 models. For model selection, we used the heuristic method used by (28), aiming to select a stable and well-fitted model. First, we selected the top ten models in terms of their posterior distribution of the relevant parameters. Among these ten models, we subsequently evaluated the agreement of view memberships between models using the Adjusted Rand Index (ARI) (34). Then, we identified a pair of models that gave the largest value of ARI. The final model was the one in this pair, which gave a larger posterior value. To regularize the correlation matrices, we simply added a small fraction (0.05) to the diagonal elements and subsequently converted it into a correlation matrix.

As a reference method for clustering, we also performed an FC-based multiple clustering method (27, 35), in which a connectivity matrix was vectorized, and each FC was considered a feature (Figure 1B). The vectorized FCs were then partitioned into views by fitting to Gaussian mixture models, in which the number of views and clusters were automatically determined in a data-driven manner.

### 2.2. Data

We used two resting-state FC datasets that are publicly available at the Strategic Research Program for Brain Sciences (SRPBS)^1^, in which FC was evaluated in a conventional manner using Pearson's correlation coefficient for mean blood-oxygen-level-dependent signals between two ROIs. These two datasets were collected at the University of Tokyo (hereafter referred to as “UTO”) and Kyoto University (hereafter referred to as “KYO”), respectively. The FC dataset of the UTO was obtained using the same MRI scanner, while the KYO dataset was obtained using two different MRI scanners. Hence, we further divided the KYO data into two datasets according to the scanner type: “KYO-A” and “KYO-B.” Detailed information on MRI scanning for UTO and KYO is provided in Table 1. Regarding brain parcellation, the BAL atlas, which is a composite of the BrainVisa Sulci Atlas (BSA) (36) and automated anatomical labeling (AAL) atlas (37) with 140 ROIs [for more details, please refer to (38)], was used for both UTO and KYO.

**Table 1:** MRI scanning information for UTO (the University of Tokyo) and KYO (Kyoto University) data.

**Configuration**	**UTO**	**KYO-A**	**KYO-B**
Scanner type	GE MR750W	Siemens trimtrio	Siemens trio
Magnetic field strength (T)	3.0	3.0	3.0
Field of view (mm)	212 × 212	212 × 212	256 × 192
Matrix	64 × 64	64 × 64	64 × 48
Number of slices	40	40	30
Number of volumes	240	240	177
	(+4 extra volumes		
	for dummy scan)		
In-plane resolution (mm)	3.3 × 3.3	3.3125 × 3.3125	4.0 × 4.0
Slice thickness (mm)	3.2	3.2	4.0
Slice gap (mm)	0.8	0.8	0
TR (ms)	2,500	2,500	2,000
TE (ms)	30	30	30
Total scan time (mm:ss)	10:00	10:00	6:00
Flip angle (deg)	80	80	90
Slice acquisition order	Ascending	Ascending	Ascending
			(interleaved)

*In addition, the instructions for participants and other imaging conditions were as follows: For UTO, “Please relax. Do not think of anything in particular. Do not sleep, but keep looking at the crosshair mark presented.;” for both KYO-A and -B, “Please relax. Fixate on the central crosshair mark and do not think of anything during rest.” The lights in the scan room were dimmed*.

The UTO dataset consisted of 322 subjects: 170 HC, 62 MDD, 41 BD, 35 SCZ, 10 ASD, and 4 dysthymia (DY) subjects, respectively (Table 2). The KYO-A dataset consisted of 219 subjects: 159 HC, 16 MDD, and 44 SCZ. The KYO-B dataset consisted of 119 subjects: 75 HC and 44 SCZ. We used the UTO data as discovery data, where cluster analysis was performed using the ROI-based multiple clustering method, and used the KYO-A and KYO-B datasets as validation data to examine the reproducibility of the UTO clustering results. In addition, we used the dataset of traveling subjects (TS) as validation data. The SRPBS depository included FC datasets for nine HC subjects who underwent fMRI scanning at different sites or with different scanners. These subjects were scanned three times for the UTO and KYO-A scanners. There was no overlapping of subjects observed among the UTO, KYO, and TS datasets.

**Table 2:** Psychiatric and demographic information of subjects in UTO (The University of Tokyo) and KYO (Kyoto University) datasets.

**Dataset**	**Diagnosis**	**Sample size**		**Sex**	**Age**	
		***N***	**%**	**M/F**	**Mean**	**Std**
UTO	HC	170	52.8	78/92	35.5	17.4
(discovery)	MDD	62	19.2	36/26	38.7	11.6
	BD	41	12.7	26/15	34.2	9.1
	SCZ	35	10.8	23/12	31.6	10.3
	ASD	10	3.1	9/1	37.0	9.5
	DY	4	1.2	3/1	30.2	16.1
	Sum	322	100	175/147	35.5	14.7
KYO-A	HC	159	72.6	93/66	36.5	13.5
(validation)	MDD	16	7.3	10/6	42.5	12.4
	SCZ	44	20.1	20/24	41.2	10.9
	Sum	219	100	123/96	37.9	13.1
KYO-B	HC	75	63.0	48/27	28.8	9.0
(validation)	SCZ	44	37.0	25/19	37.2	9.6
	Sum	119	100	73/46	31.9	10.1

*HC, healthy control; MDD, major depressive disorder; BD, bipolar disorder; SCZ, schizophrenia; ASD, autism spectrum disorder; DY, dysthymia*.

## 3. Results

First, we performed cluster analysis for the UTO data by fitting the ROI-based multiple clustering method. Second, to verify the clustering results of the UTO data, we classified the subjects in the KYO data based on the statistical model inferred from the UTO data. For further verification, we classified the subjects in the TS dataset based on the UTO model. Finally, for the purpose of comparison, we performed a supplementary analysis of the UTO data using the supervised learning method.

### 3.1. Discovery Data

We applied the ROI-based multiple clustering method to UTO data. For comparison with the clustering results, we also applied the FC-based multiple clustering method accordingly.

#### 3.1.1. Cluster Solutions

The ROI-based multiple clustering method yielded 15 views (number of ROIs, 2–25; number of clusters, 3–77; Table 3). In this analysis, we assigned view labels and cluster labels as follows. Views were sorted in ascending order of the number of subject clusters in a view, whereas clusters in each view were sorted in a descending order of the number of subjects. The FC-based multiple clustering method yielded 13 views (number of FCs, 26–2,830; number of clusters, 5–10). Views and clusters were sorted in the same manner as the results of the ROI-based multiple clustering method.

**Table 3:** Summary of the multiple clustering results.

**ROI-based method**			**FC-based method**		
**View ID**	**Number of**	**Number of**	**View ID**	**Number of**	**Number of**
	**ROIs**	**clusters**		**FCs**	**clusters**
1	2	3	1	161	5
2	3	5	2	128	5
3	25	7	3	53	5
4	15	12	4	26	5
5	7	13	5	126	6
6	9	13	6	325	7
7	7	14	7	421	8
8	14	14	8	340	8
9	11	16	9	2,820	9
10	9	19	10	1,238	9
11	6	28	11	774	9
12	6	29	12	2,830	10
13	9	36	13	488	10
14	8	60			
15	9	77			
Sum	140			9,730	

*The view ID is sorted in ascending order of the number of clusters for each method*.

**Figure 2:**
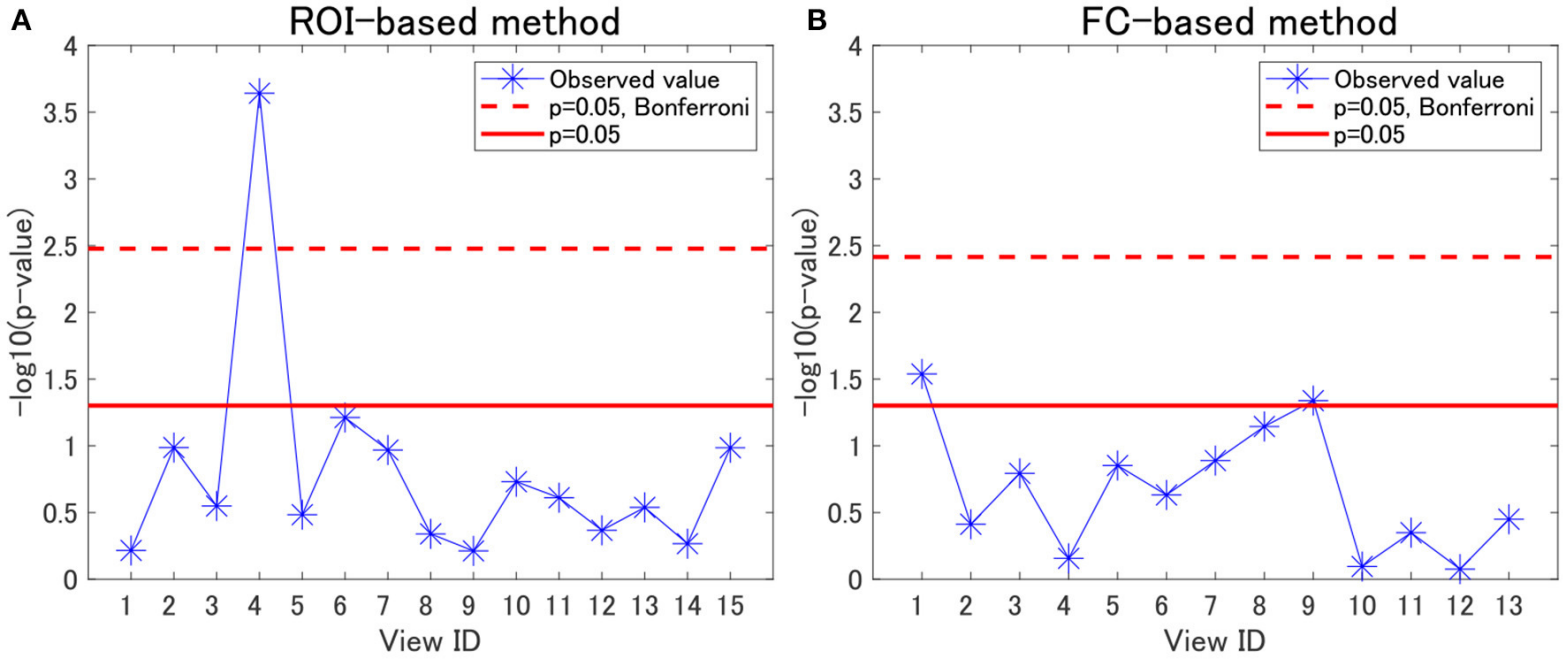
Associations between cluster labels and psychiatric disorders. **(A)** Results of the ROI-based multiple clustering method. **(B)** Results of the FC-based multiple clustering method. The horizontal axis denotes the view ID, whereas the vertical axis denotes the negative logarithm of the *p*-value yielded by the χ^2^ test to evaluate the association between the corresponding cluster labels in the view and psychiatric labels. The red line denotes the significance level at 0.05, whereas the dotted line denotes the significance level at 0.05, with Bonferroni correction.

For each view, we evaluated the association between cluster labels and psychiatric disorders, including HC. For the ROI-based multiple clustering method, the subject clusters in view 4 were significantly associated with psychiatric disorders, as shown by the Pearson's χ^2^ test for contingency tables (simply referred to as “χ^2^ test” hereafter) (*p* = 0.0002, significant at the 0.05 level with Bonferroni correction; Figure 2A). In contrast, for the FC-based multiple clustering method, no significant association was found with the Bonferroni correction (Figure 2B). Therefore, for further analysis, we focused on view 4 yielded by the ROI-based multiple clustering method (we discuss the remainder of the views in section 4.1).

**Figure 3:**
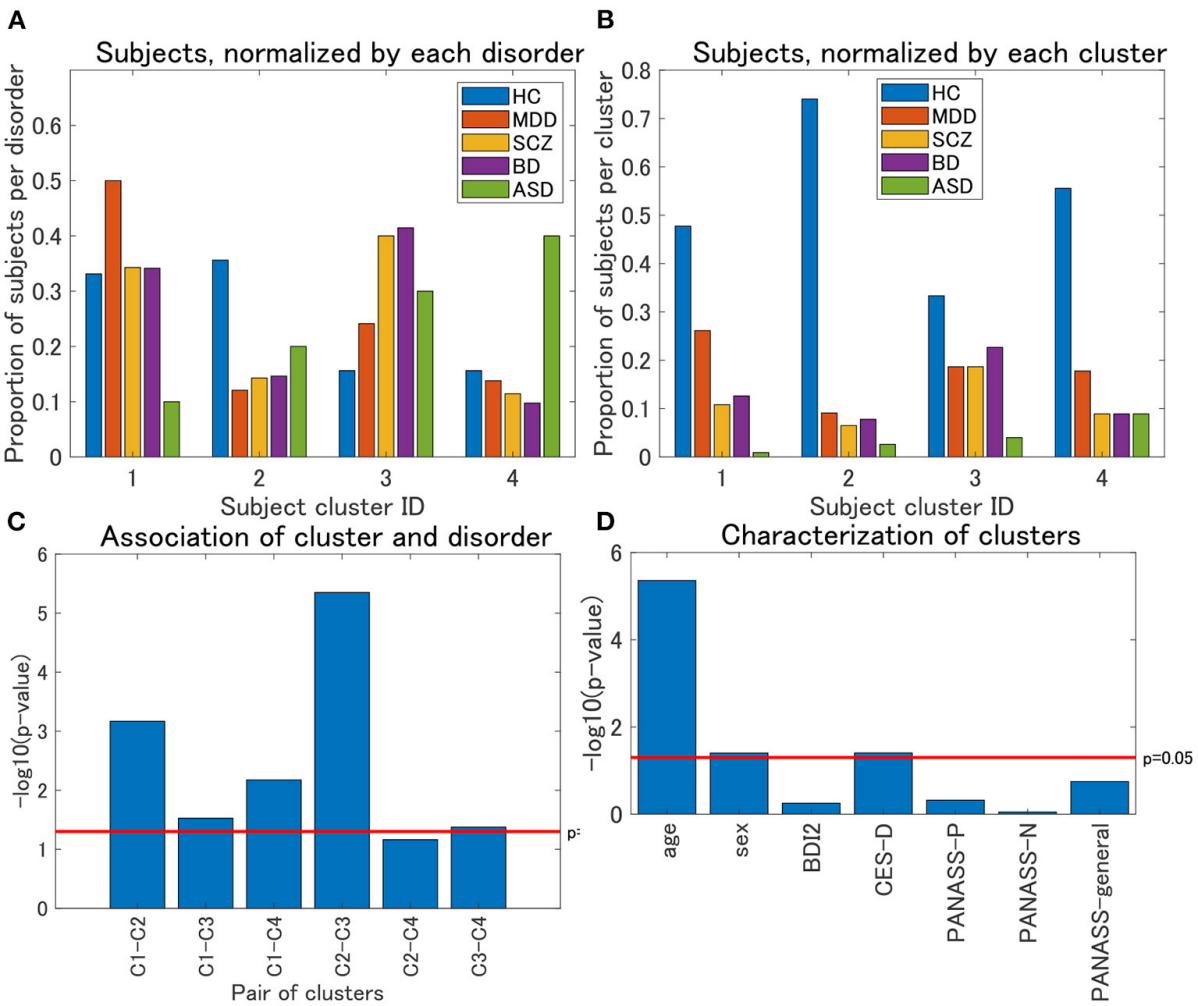
**(A)** Distribution of subjects of UTO data for view 4, normalized by each disorder. The horizontal axis denotes the cluster ID, whereas the vertical axis denotes the proportion of subjects for each psychiatric disorder over all the four clusters. For each cluster, the proportion of subjects with a particular psychiatric disorder is denoted by a colored bar. Note that the proportion is evaluated in the disorder-wise manner. That is, the summation of the four proportions becomes one for each disorder (e.g., for HC, the summation of the proportions denoted by blue bars becomes one). Furthermore, note that clusters with <10 subjects were removed. **(B)** Distribution of subjects of UTO data for view 4, normalized by each cluster. In contrast with **(A)**, the proportion of subjects is evaluated in a cluster-wise manner. That is, the summation of the five proportions becomes one for each cluster (e.g., for cluster 1, the summation of the proportions denoted in blue, red, orange, purple, and green becomes one). **(C)** Results of the χ^2^ test for the association between pairs of cluster labels and pairs of psychiatric labels in view 4. For the pairs of psychiatric labels, we consider those psychiatric labels that characterize the pair of clusters in question. Namely, MDD and HC for clusters 1 and 2, MDD and SCZ/BD for clusters 1 and 3, MDD and ASD for clusters 1 and 4, HC and SCZ/BD for clusters 2 and 3, HC and ASD for clusters 2 and 4, and SCZ/BD and ASD for clusters 3 and 4. The horizontal axis denotes the pair of cluster labels, whereas the vertical axis denotes the negative logarithm of the p-value by the χ^2^ test. **(D)** Characterization of four clusters in view 4. The horizontal axis denotes demographic/clinical indices, whereas the vertical axis denotes the negative logarithm of the *p*-value by the χ^2^ test on cluster labels and sex, and the Kruskal-Wallis test on cluster labels and the remainder of the indices. BDI2, beck depression inventory II; CES-D, center for epidemiologic studies depression scale; PANASS-P, positive and negative syndrome scale consisting of positive psychopathology scale; PANASS-N, negative scales; PANASS-general, general scales.

Regarding view 4, we first examined the distribution of psychiatric labels in the clusters. In this view, 12 clusters were yielded, with sample sizes of 111, 77, 75, 45, 4, 4, 1, 1, 1, 1, 1, and 1 for clusters 1–12, respectively (Table 4). For further analysis, we focused on clusters 1–4, which had sample sizes larger than 10. To alleviate the imbalanced distribution of psychiatric labels in the data, we evaluated the proportions of subjects in the disorder-wise manner (Figure 3A), which showed that the subject distribution of these clusters was closely associated with psychiatric labels. In contrast, the proportions of subjects evaluated in a cluster-wise manner reflected the imbalanced distribution of psychiatric labels in the data (Figure 3B). For a better understanding of clusters, hereafter, we deal with the proportions of subjects in a disorder-wise manner, as in Figure 3A. Based on the disorder-wise proportions of subjects, we characterized these clusters in terms of the proportion of each psychiatric label over the clusters as follows: cluster 1, MDD; cluster 2, HC; cluster 3, SCZ/BD; and cluster 4, ASD. Note that we combined SCZ and BD because their subject distributions were similar for clusters 1–4; for any pair of clusters, there was no difference noted in the distributions for the two psychiatric labels using the χ^2^ test.

**Table 4:** Distribution of psychiatric disorders of UTO data for clusters in view 4.

**Cluster ID**	**HC**	**MDD**	**SCZ**	**BD**	**ASD**	**DY**	**Sum**
1	53	29	12	14	1	2	111
2	57	7	5	6	2	0	77
3	25	14	14	17	3	2	75
4	25	8	4	4	4	0	45
5	4	0	0	0	0	0	4
6	3	1	0	0	0	0	4
7	0	1	0	0	0	0	1
8	0	1	0	0	0	0	1
9	1	0	0	0	0	0	1
10	1	0	0	0	0	0	1
11	1	0	0	0	0	0	1
12	0	1	0	0	0	0	1
Sum	170	62	35	41	10	4	322

*The digits in the table denote the number of subjects*.

For each pair of clusters, we performed the χ^2^ test on the association between the cluster and psychiatric labels. To this end, we focused on specific psychiatric disorders that characterized the pairs of clusters in question. For instance, to test the pair of clusters 1 and 2, we considered the psychiatric labels of MDD and HC only because these psychiatric labels characterize clusters 1 and 2, respectively. We found that these associations were significant at a level of 0.05, for any pair of clusters (Figure 3C), except for the pair of clusters 2 and 4 (*p* = 0.069), which supported the aforementioned characterization of the four clusters.

We also characterized the subject clusters using demographic and clinical information. It was found that age was significantly related to these clusters (*p* = 4.4 × 10^−6^; Figure 3D). The mean age is at 36.4, 29.5, 35.8, and 40.9 years for clusters 1–4, respectively (Supplementary Figure 1A), showing that it is rather small for cluster 2. Moreover, we examined the association between age and psychiatric disorders. For HC, the age difference between cluster 2 and the remaining three clusters was significant (Kruskal–Wallis test, p = 0.0003), whereas such differences were not significant for MDD, SCZ, and BD (*p* = 0.21, 0.39, and 0.10, respectively; Supplementary Figures 1B–D).

To summarize, the results of the analysis of the four clusters suggest that we may characterize the clusters in view 4 as follows:

- Cluster 1: MDD
- Cluster 2: young HC
- Cluster 3: SCZ/BD
- Cluster 4: ASD

where “young HC” denotes the HC subjects of a relatively young age (around 20 years).

For the characterization of the clusters, it is noteworthy that HC (as well as the remainder of the psychiatric disorders) is, to some extent, included in all clusters. One may wonder whether there is a difference in depression scores (BDI or CES-D) for HC between these clusters. For BDI, the difference among clusters was not significant (Kruskal–Wallis test, *p* = 0.58), whereas for CES-D, the difference was significant (*p* = 0.016). Furthermore, for CES-D, the pairwise test for these clusters suggests that the difference was significant for the following pairs of clusters: cluster 4 < cluster 3 (*p* = 0.0044), cluster 4 < cluster 2 (*p* = 0.022), and cluster 1 < cluster 3 (*p* = 0.048). In particular, this result provides additional characterization for Cluster 4 as a non-depressive disorder. We discuss the interpretation of this result in section 4.3.

#### 3.1.2. Relevant Brain Region

Furthermore, we examined the relevant brain regions for the four clusters in view 4. The subnetwork for view 4 consisted of a cerebellum-thalamus-pallidum-temporal circuit. The relevant ROIs for this subnetwork are as follows: left posterior intra-lingual sulcus; right anterior occipito-temporal lateral sulcus; left median occipito-temporal lateral sulcus; right median occipito-temporal lateral sulcus; left posterior occipito-temporal lateral sulcus; right anterior inferior temporal sulcus; left posterior inferior temporal sulcus; right posterior inferior temporal sulcus; right polar temporal sulcus; left superior temporal sulcus; left thalamus; left pallidum; right thalamus; left cerebellum; and vermis.

**Figure 4:**
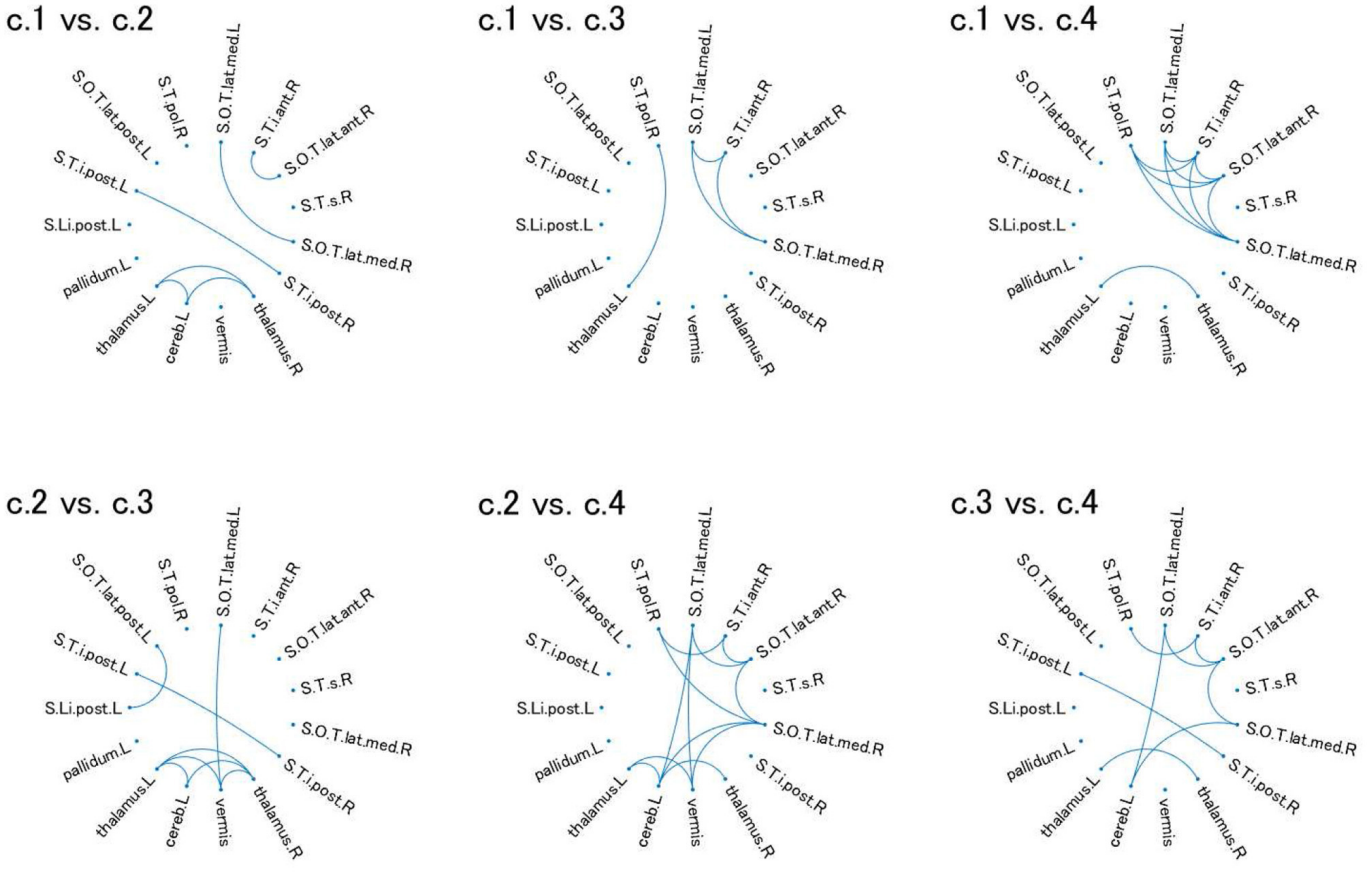
Relevant connectivity for differences between pairs of the four clusters in view 4. The relevant connectivity for the difference between cluster *i* and cluster *j* is displayed in panel “c.*i* vs. c.*j*.” The relevance of connectivity is evaluated by Cohen's *d* for the effect size of FC differences between two clusters. The connectivity with *d* > 0.8 [large separability; (39)] is shown in the diagram in which the ROI is denoted as a dot. S.Li.post.L, left posterior intra-lingual sulcus; S.O.T.lat.ant.R, right anterior occipito-temporal lateral sulcus; S.O.T.lat.med.L, left median occipito-temporal lateral sulcus; S.O.T.lat.med.R, right median occipito-temporal lateral sulcus; S.O.T.lat.post.L, left posterior occipito-temporal lateral sulcus; S.T.i.ant.R, right anterior inferior temporal sulcus; S.T.i.post.L, left posterior inferior temporal sulcus; S.T.i.post.R, right posterior inferior temporal sulcus; S.T.pol.R, right polar temporal sulcus; S.T.s.R, left superior temporal sulcus; thalamus.L, left thalamus; pallidum.L, left pallidum; thalamus.R, right thalamus; cereb.L, left cerebellum; vermis, vermis. For visualization of these brain regions, see Supplementary Figure 2.

**Figure 5:**
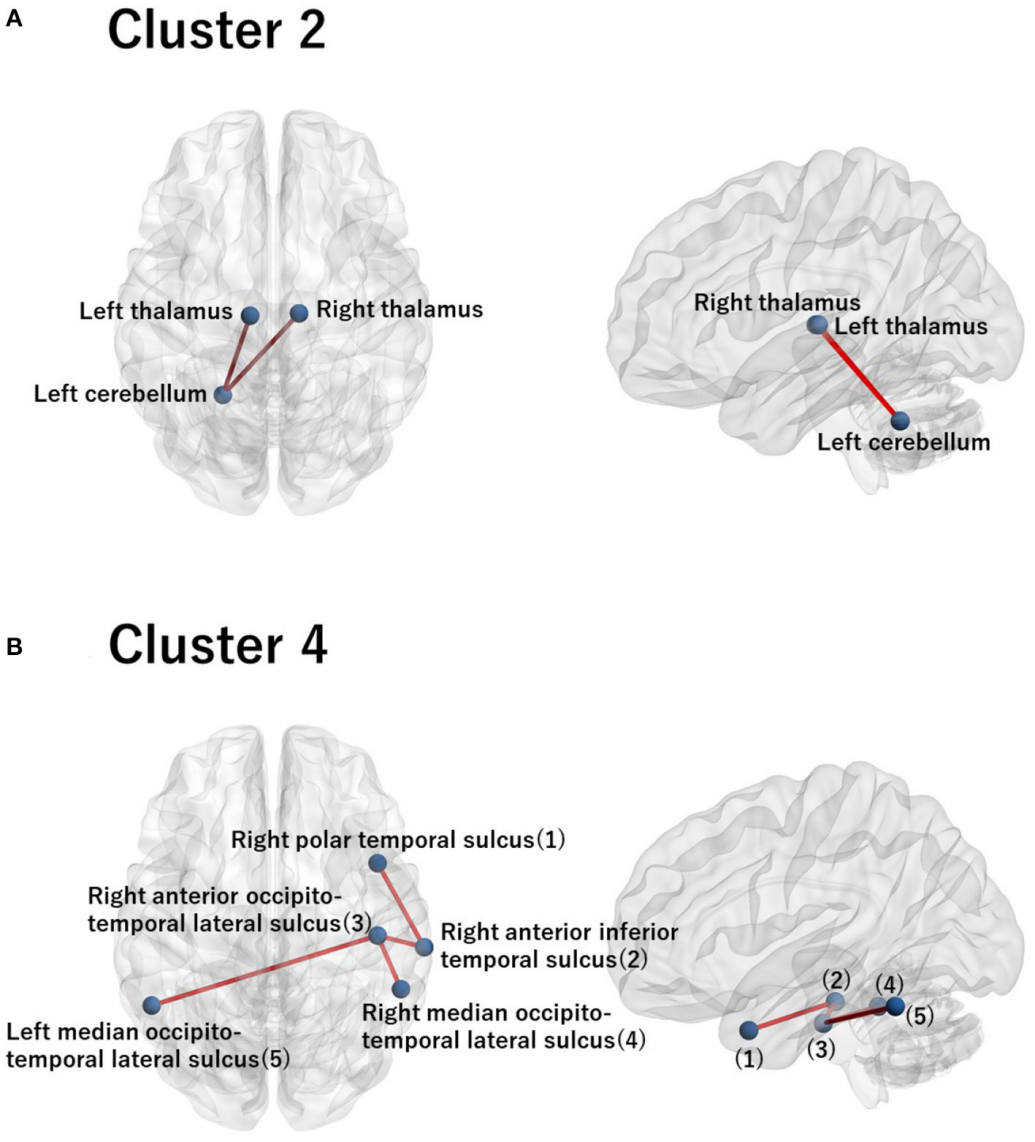
Visualization of the commonly important FC for cluster 2 **(A)** and for cluster 4 **(B)**. **(A)** The commonly important FC that discriminates cluster 2 against the remainder of the clusters with the criterion Cohen's *d* > 0.8: right thalamus−left cerebellum−left thalamus. **(B)** The commonly important FC that discriminates cluster 4 against the remainder of the clusters with the criterion Cohen's *d* > 0.8: right polar temporal sulcus−right anterior inferior temporal sulcus−right anterior occipito-temporal lateral sulcus− (left and right) median occipito-temporal lateral sulcus. The numbering in the sagittal image denotes the corresponding ROI names in the axial image. Note that there is no commonly important FC for clusters 1 and 3 with the criterion Cohen's *d* > 0.8. Hence, we did not visualize brain images for these clusters.

For these ROIs, we identified FCs that were specifically relevant to a pair of clusters. To this end, we evaluated Cohen's *d* (39) for FC differences between the pairs of clusters. We found that several FCs largely discriminate between two clusters, following the conventional criterion of *d* > 0.8 (Figure 4, Supplementary Figure 2). Moreover, to narrow down to an individual cluster, we examined the commonly important FC in Figure 4 for a particular cluster against the remainder of the clusters. We found that clusters 2 and 4 have a common important FC, as shown in Figure 5:

- Cluster 2: right thalamus−left cerebellum−left thalamus
- Cluster 4: right polar temporal sulcus−right anterior inferior temporal sulcus−right anterior occipito-temporal lateral sulcus−(left and right) median occipito-temporal lateral sulcus.

We discuss the interpretation of these results in more detail in section 4.2.

### 3.2. Validation Data

In this section, we examine the reproducibility of the clustering results from view 4. Here, we classified the subjects of two independent datasets, KYO-A and KYO-B, using the clustering model in view 4. Furthermore, we also classified subjects in the TS data to examine the reproducibility of classification at the individual subject level.

#### 3.2.1. KYO Data

We examined the validity of the clustering results from view 4, which was obtained in the previous section. We classified KYO subjects based on the UTO model (Table 5). Note that the KYO data were not used for model estimation; hence, they were independent of the estimated model. For pre-processing, we separately applied the whitening procedure for KYO-A and KYO-B (referred to as “KYO-whitening”). Subsequently, the classification was performed for each subject by fitting the Wishart mixture model of view 4 to the subject correlation matrix. To compare the performance, a similar classification was also performed for the KYO datasets that were whitened using the UTO data (referred to as “UTO-whitening”).

**Table 5:** Summary of classification of KYO subjects for view 4 with KYO-whitening and UTO-whitening.

	**KYO-whitening**					**UTO-whitening**				
**Cluster**	**KYO-A**			**KYO-B**		**KYO-A**			**KYO-B**	
**ID**	**HC**	**MDD**	**SCZ**	**HC**	**SCZ**	**HC**	**MDD**	**SCZ**	**HC**	**SCZ**
1	57	6	12	22	16	35	5	14	28	25
2	46	2	6	21	5	80	6	8	20	4
3	31	6	18	17	14	13	1	6	7	5
4	21	2	5	9	4	22	3	9	8	4
5	1	0	0	1	0	1	0	1	2	0
6	0	0	1	1	0	0	0	0	1	0
7	0	0	0	0	0	0	0	0	0	0
8	0	0	0	0	0	0	0	0	0	0
9	0	0	0	0	0	0	0	0	0	0
10	0	0	0	1	0	0	0	0	1	0
11	1	0	0	1	0	1	1	0	0	0
12	0	0	0	0	0	0	0	0	0	0
New	2	0	2	2	5	7	0	6	8	6
Sum	159	16	44	75	44	159	16	44	75	44

*The classification is obtained by fitting the ROI-based multiple-clustering method to each KYO subject using the estimated parameters yielded from the UTO data. Note that the ROI-based multiple-clustering method determines the number of clusters in a data-driven manner. Owing to this nature of the method, a subject can be allocated to a new cluster that consists of itself. In such a case, the subject is considered to be allocated to a “new” cluster*.

**Figure 6:**
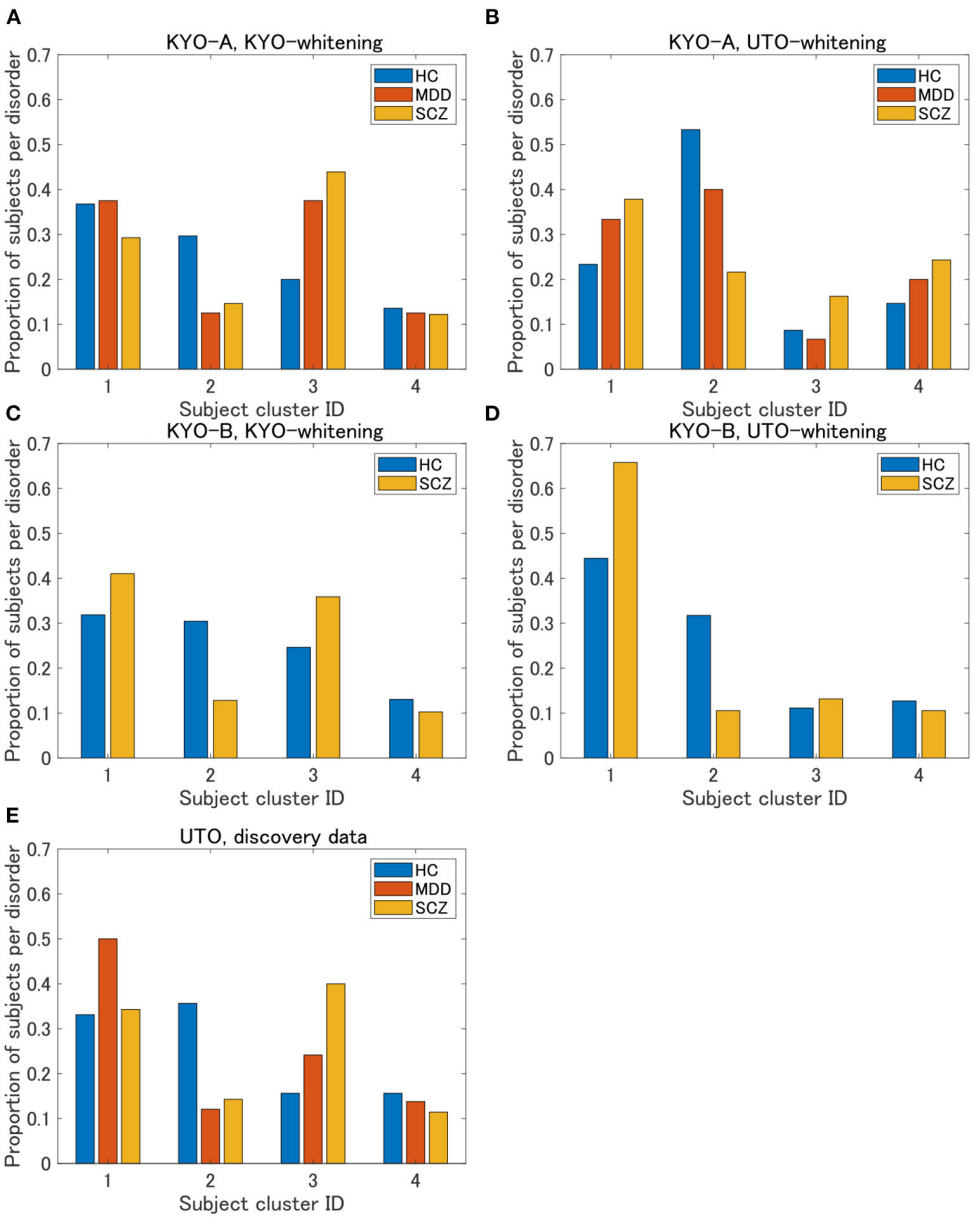
Results of validation for KYO data. **(A,B)** Proportion of subjects per disorder of KYO-A data with KYO-whitening and with UTO-whitening, respectively. **(C,D)** Similar graphs for KYO-B data. **(E)** Proportion of subjects per disorder of UTO data. This is a copy of Figure 3A, but to compare with the results of the KYO data, the proportion is displayed only for HC, MDD, and SCZ.

To examine the reproducibility of the clustering results, we consider psychiatric labels and age distributions in the study sample. First, a visual inspection suggests that for KYO-whitening, the psychiatric label distribution of subjects over clusters is quite similar between KYO-A/B and UTO (Figures 6A,C,E), whereas this is not the case for UTO-whitening (Figures 6B,D,E). More precisely, focusing on HC, SCZ, and MDD (MDD is applicable only for KYO-A), the χ^2^ test for the difference in the subject distribution between KYO and UTO supports this observation (Table 6). For KYO-whitening, the difference between KYO-A and UTO was not significant for HC, SCZ, or MDD (*p* = 0.52, 0.97, and 0.73, respectively). Similarly, the difference between KYO-B and UTO was not significant for either HC or SCZ (*p* = 0.43 and 0.94, respectively). In contrast, for UTO-whitening, the difference between KYO-A and UTO was significant for HC and MDD (*p* = 0.010 and 0.045, respectively) but not for SCZ (*p* = 0.11). Furthermore, the difference between KYO-B and UTO was significant for SCZ but not for HC (*p* = 0.031 and *p* = 0.44, respectively). Next, we evaluated the extent of the difference between two clustering results by means of Cramér's *V* (40, 41) (Table 6). For KYO-whitening, the average Cramér's *V* is 0.09 for both KYO-A and KYO-B, whereas for UTO-whitening it is 0.27 and 0.23 for KYO-A and KYO-B, respectively. Following Cohen's criterion for effect size *V* (equivalent to Cohen's w: small 0.10; medium 0.30; large 0.50) (39), this result suggests that the clustering difference is small for KYO-whitening, whereas for UTO-whitening, the difference is medium.

**Table 6:** Differences of clustering results (clusters 1–4 in view 4) between the discovery and validation data based on the χ^2^ test.

	**KYO-whitening**				**UTO-whitening**			
	**KYO-A**		**KYO-B**		**KYO-A**		**KYO-B**	
	**V**	***p*-value**	**V**	***p*-value**	**V**	***p*-value**	**V**	***p*-value**
HC	0.084	0.52	0.11	0.43	0.19	0.010*	0.11	0.44
MDD	0.13	0.73	N/A	N/A	0.33	0.045*	N/A	N/A
SCZ	0.055	0.97	0.069	0.94	0.29	0.11	0.35	0.031*
Average	0.090		0.090		0.27		0.23	

Instead of the χ^2^ statistic, we report on Cramér's V (0 ≤ V ≤ 1), which is defined as $\sqrt{\chi^{2}/N}$ with a sample size N. Cramér's V is evaluated for a contingency table of clustering results for each disorder and each type of validation data. If there is a large difference between the UTO and the corresponding KYO data, V takes a value close to one. If there is no difference, V approaches zero. Cramér's V is displayed in the first column for each type of dataset, whereas in the second column, the p-value is displayed (^*^p < 0.05).

Regarding age distribution, we then examined whether the age difference among clusters for the UTO data was reproduced for the KYO data. For both KYO- and UTO-whitening, the age of the subjects in cluster 2 was relatively small (Supplementary Figure 3); however, the age difference among the clusters was minor. More precisely, for KYO-whitening, the difference was not significant for KYO-A and KYO-B (Kruskal–Wallis test; *p* = 0.41, and *p* = 0.53, respectively), whereas for UTO-whitening, the difference was significant for KYO-A but not for KYO-B (Kruskal–Wallis test; *p* = 0.0034 and *p* = 0.36, respectively).

#### 3.2.2. Traveling Subject Data

**Figure 7:**
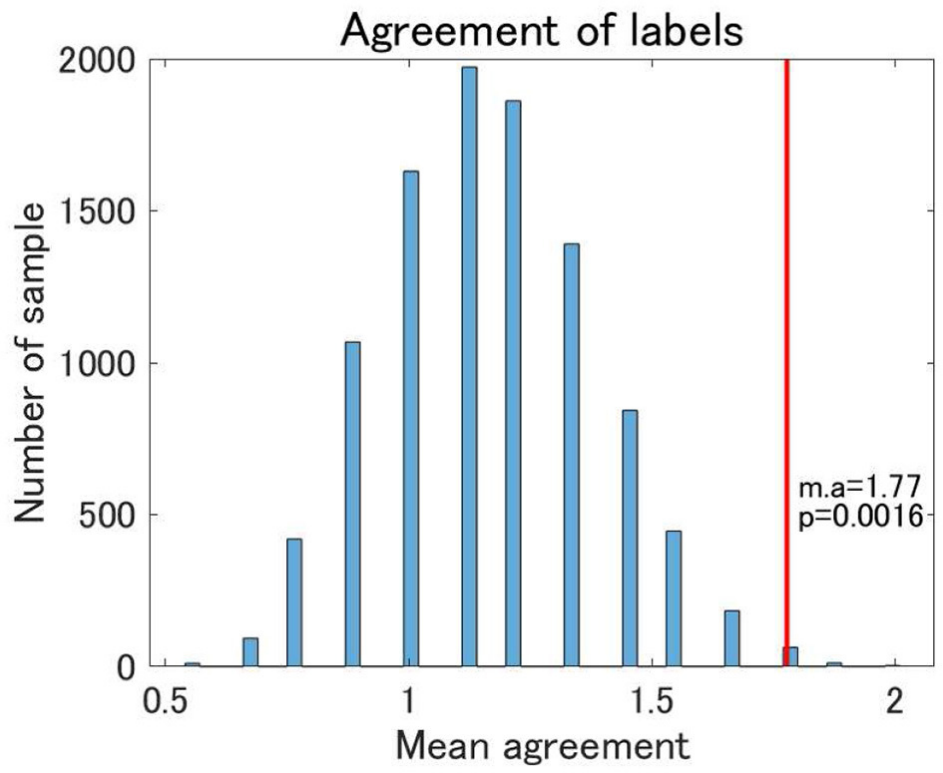
Agreement of cluster labels of traveling subjects (TS) between UTO scanner and KYO-A scanner. The agreement was measured as follows: each traveling subject performed three repetitions of fMRI scans for both the UTO scanner and KYO-A scanner. For each scan, we evaluated the FC matrix, which was subsequently used for classification based on the estimated model of view 4. Next, we evaluated the number of scans out of the three agreements between UTO and KYO-A (minimum 0; maximum 3). Finally, we took the average of the agreement for all traveling subjects (nine subjects). The null distribution of agreement is shown in the bar chart, which is based on 10,000 randomly shuffled TS data. The red line denotes the observed value of the mean agreement (the observed mean agreement and *p*-value are displayed on the right). Note that the correlation matrices are whitened by the corresponding datasets.

Finally, we examined the reproducibility of the view 4 clustering results using TS data. We found that the reproducibility of cluster labels at the individual level was rather limited, with some variations in cluster labels observed for three scans at the same site (Supplementary Table 1). Nonetheless, focusing on the pattern of cluster labels, the reproducibility at the individual level was statistically significant between UTO and KYO-A using the permutation test (Figure 7). Furthermore, at the group level, the cluster-wise distribution of the total number of subjects was similar between UTO and KYO-A (Supplementary Table 2; *p* = 0.26, using the χ^2^ test).

### 3.3. Supervised Classification

The framework of our analysis has so far been unsupervised learning, without explicitly using psychiatric label information. We used the label information only when we characterized the clustering results, which showed a correspondence between the yielded clusters and psychiatric disorders. One may wonder whether such a correspondence may become clearer in the framework of supervised learning, explicitly using the label information for model development. To address this issue, as a supplementary analysis, we performed a supervised classification of the UTO data. For simplicity, we based our multiclass classification on a pairwise classification. First, we created a classification model for each pair of five psychiatric disorders (HC, MDD, SCZ, BD, and ASD) in a supervised manner. In so doing, we balanced the sample size for the corresponding psychiatric disorders by subsampling subjects with psychiatric disorders with a larger sample size. For this balanced data, we evaluated the classification probability of the subjects in the data in a framework of leave-one-out cross-validation. Subsequently, we created a classification model using all the subjects in this balanced dataset. Second, using the classification model, we evaluated the classification probabilities of the remaining subjects. In this classification method, all subjects were classified as the test data. We then repeated this procedure for all pairs of psychiatric disorders, which yielded a vector of classification probability of the pair of psychiatric disorders for each subject. Therefore, for subject *i*, we obtained a classification probability *p*_*i*_(*j, k*) (*j* ≠ *k*), which denoted the probability that subject *i* belonged to a psychiatric disorder *j* in the classification model of that psychiatric disorder *j* vs. psychiatric disorder *k*. Note that *p*_*i*_(*j, k*) = 1 − *p*_*i*_(*k, j*). Third, for each subject *i*, we evaluated the marginal classification probability for a particular psychiatric disorder *j* by averaging the classification probabilities *p*_*i*_(*j, k*) over psychiatric disorders *k*. Finally, we assigned a classification label to each subject based on the marginal classification probability (i.e., the label with the largest marginal probability). For the pairwise classification, we applied elastic net classification to vectorized FC data, which is a linear classification method with *L*_1_ and *L*_2_ regularization (42). We considered two pre-processing steps: regression-out and non-regression-out of age and sex effects from the data.

**Figure 8:**
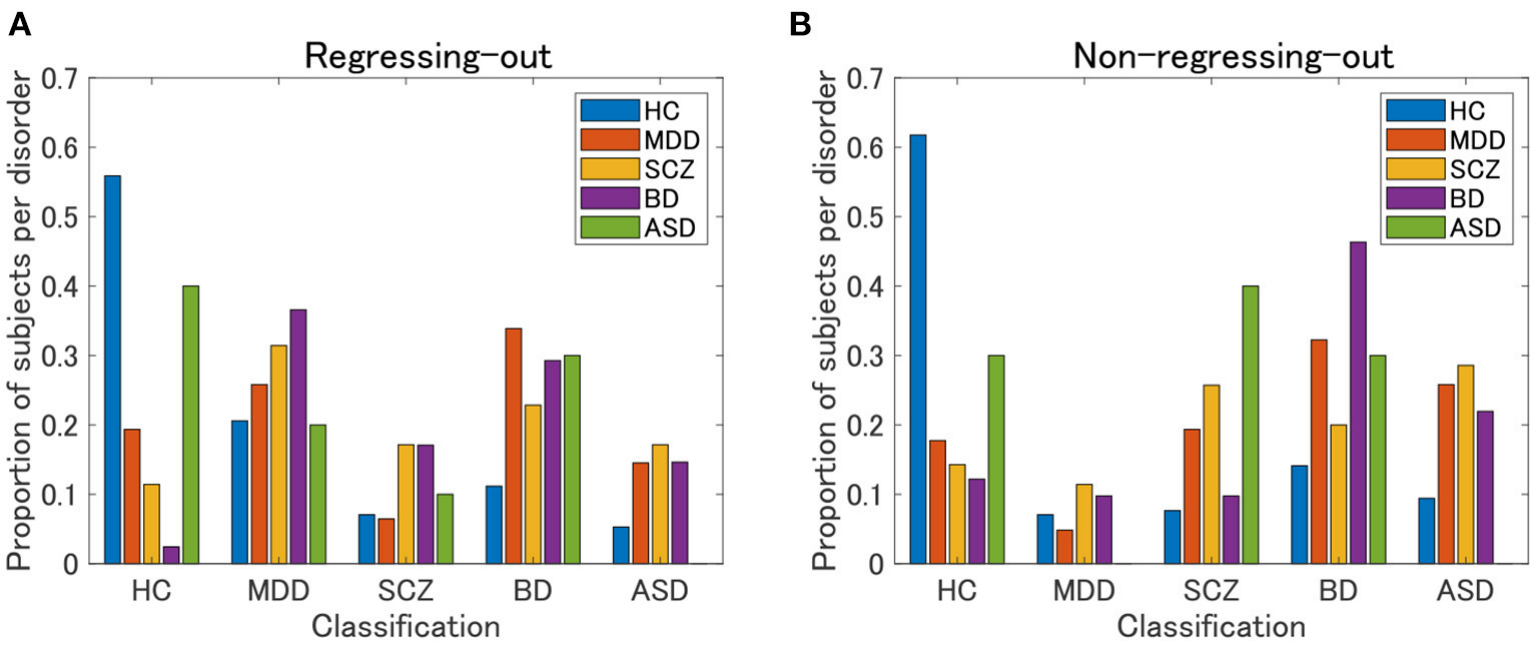
Classification results of UTO subjects in the supervised approach. First, a classification model is built for each pair of psychiatric disorders, including HC, by applying elastic net classification to vectorized FC data. Second, based on the marginal probability yielded by these classification models, a subject is classified into one of the psychiatric categories. **(A)** FC is pre-processed by regressing-out of the effect of age and sex. **(B)** FC is not preprocessed.

The HC classification worked well for both the regression-out and non-regression-out cases (Figure 8, Supplementary Table 3). However, the performance of the classification of psychiatric disorders was rather poor, except for BD in the non-regression-out case, in which the majority of BD subjects were correctly classified into the BD category. Further, we evaluated the agreement between the classification results and the clustering results (clusters 1–4 in view 4) by means of ARI. For the regression-out and non-regression-out cases, ARI was 0.036 (*p* = 1.4×10^−4^ in the permutation test) and 0.054 (*p* = 6.0×10^−6^), respectively. When we excluded HC, the ARI was 0.022 (*p* = 0.048) and 0.025 (0.028), respectively. This suggested that there might be a small correspondence between the supervised and unsupervised results.

## 4. Discussion

### 4.1. Clustering Results

The ROI-based multiple clustering method revealed four clusters in view 4 of the UTO data that were characterized by psychiatric disorders: cluster 1 by MDD, cluster 2 by young HC, cluster 3 by SCZ/BD, and cluster 4 by ASD. The difference in psychiatric label distributions between a pair of clusters was significant when we focused on psychiatric disorders characterizing the clusters in question (except for the pair of clusters 2 and 4). The relevant subnetwork for these clusters consisted of 15 ROIs in a cerebellum-thalamus-pallidum-temporal circuit, which may suggest common functional connectivity to discriminate between HC, MDD, SCZ/BD, and ASD.

Regarding cluster 2 and the age effect, the statistical test showed that the age difference among clusters was significant for HC, whereas this was not the case for the remainder of the psychiatric disorders. This suggested that the effect of age was limited to HC only. Accordingly, it is worth noting that in Cluster 2, the proportion of psychiatric disorders was rather small. Hence, we can interpret that the FC pattern of the majority of psychiatric disorders is largely different from that of young HCs.

For cluster 3, note that SCZ and BD were not discriminated in the present study because there was no difference in the distributions between SCZ and BD over clusters using the χ^2^ test. This result is consistent with the growing evidence in the literature for phenological, biological, and genetic overlaps between SCZ and BD (43, 44).

The association between the four clusters and psychiatric disorders was largely reproduced by the validation datasets. First, the distribution pattern of psychiatric labels for the UTO data was reproduced for HC, SCZ, and MDD of KYO-A data and for HC and SCZ of KYO-B data with KYO-whitening. Regarding the age difference between clusters, the same tendency was observed between the UTO and KYO data, although it was not statistically significant. Moreover, reproducibility was not obtained when we inappropriately whitened the KYO data using the UTO data, which further strengthened the validity of the classification results with KYO-whitening. Nonetheless, the reproducibility discussed here is limited to the sense of grouped data since the subjects differed between the discovery and validation data. The analysis of the TS data showed that even under the same conditions of the site and scanner, the classification results may differ among the three scans for a single subject. Nonetheless, the distribution pattern of cluster labels was statistically consistent at the individual subject level. This demonstrated the extent to which the clustering results were valid and the level of statistical consistency of the distribution pattern of cluster labels at the individual subject level. One possible interpretation of this result is dynamic FC, a phenomenon in which FC presumably changes dynamically (45, 46). The dynamic nature of FC may contribute to the variation in classification results for a single subject, possibly because of the insufficient number of fMRI volumes.

Finally, we discuss views other than View 4. In the present study, we mainly focused on view 4, in which the cluster labels and psychiatric labels, including HC, showed a close association. Nonetheless, this does not rule out the usefulness of the remainder of the views. An additional analysis of paired psychiatric disorders suggests that view 6 is relevant for ASD and SCZ (Supplementary Figure 4). Likewise, it is expected that the remainder of these views may have clinical and biological implications. However, because of the limited clinical information on the subjects, it is not straightforward to characterize these views in the present framework.

### 4.2. Relevant Brain Regions

Combined with the characterization of the clusters using psychiatric labels, we also interpreted clusters 2 and 4 in terms of the commonly important FC (Figure 5). First, cluster 2 was dominated by young HCs, with a small proportion of subjects with psychiatric disorders (Figure 3A). This suggests that the relevant FC in the cerebellothalamic circuit for cluster 2 is related to the contrast between young HCs and various psychiatric disorders. Several previous studies have reported on the relevance of this circuit for SCZ, which is referred to as the “cognitive dysmetria” theory (47). Cognitive dysmetria theory posits that dysfunction in this circuit impairs coordination of the mental process. A recent study (48) using two independent datasets showed that the abnormality of this circuit for SCZ is trait-dependent rather than state-dependent, which implies the underlying dysfunction of the circuit for SCZ. Furthermore, a study (49) suggested that the circuit can function as a possible biomarker for SCZ progression. In contrast, for MDD, this circuit has not been considered as a major biomarker of the disease (50). A study (51) showed that this circuit and DMN are closely associated with MDD, which is correlated with the Hamilton Depression Rating scale (51). In contrast, for BD, the role of the cerebellum in brain circuits remains unclear (52). To the best of our knowledge, there are no reports on the association between this circuit and BD, except for the recent cross-disorder study by (8). Nonetheless, for a better understanding of various psychiatric disorders, it has recently been suggested that the cerebellothalamic circuit may be added to psychomotor modulation (7, 53). The results of the present study are in line with the state-of-the-art understanding of shared neural circuits for various psychiatric disorders.

Second, cluster 4 is characterized by ASD with the relevance of FC between several temporal regions, including the occipito-temporal region. However, the important connectivity patterns for ASD remain unclear in literature (54). Nonetheless, it has been reported that the fluid intelligence of ASD is strongly associated with FC between the occipito-temporal region and the angular gyrus, posterior cingulate, and precuneus (55). Furthermore, it has been shown that FC between the occipito-temporal region and the posterior right temporo-parietal junction is correlated with social deficits in ASD (56). In conclusion, the yielded brain circuit in the present study is a new finding that discriminates ASD against HC and the remainder of the psychiatric disorders.

### 4.3. Methodology

The ROI-based multiple clustering method is unique in two ways. First, it reveals the underlying multiple-view structure in the data, which allows feature selection for a particular cluster solution. Second, it identifies the relevant subnetworks of the ROI for cluster solutions. As shown in Figure 4, these properties are useful for identifying the underlying loop or tree structure of several ROIs related to a particular cluster solution. It is expected that this novel clustering method will pioneer data-driven subnetwork analysis for psychiatric disorders. However, the FC-based multiple clustering method does not provide a useful view in the present research. A possible reason for the poor performance is the considerably high feature dimension when an FC matrix is vectorized, which hinders the effective search of the optimal solution and leads to unstable cluster solutions. In addition, it could be attributed to the vectorization of the FC matrix, which may mask the underlying useful information to discriminate between psychiatric labels.

Next, the classification results of the KYO data suggests the importance of whitening the FC matrices. The whitening procedure involves a linear transformation of correlation matrices using the sample mean correlation matrix as the benchmark, which is analogous to normalization in conventional data processing. The better performance of KYO-whitening than UTO-whitening suggests that the measurement bias attributed to the site or scanner (57) may be removed through whitening.

Furthermore, in the present study, the supervised approach using elastic net classification did not work well for the classification of psychiatric disorders. HC subjects were classified well into the correct category, whereas psychiatric patients were not. Note that most patients were classified into non-HC categories, which suggested that the supervised classifier correctly discriminated between HC and non-HC subjects. Notably, none of the subjects with ASD were correctly categorized. This was possibly due to the very small sample size of ASD subjects (*N* = 10). In contrast, our unsupervised approach was able to identify cluster 4, which was characterized by ASD. This was possibly due to the prominence of cluster 4 in the unsupervised approach, not only by ASD but also by other non-ASD subjects with FC patterns similar to those of ASD subjects. However, it is currently not clear whether the misclassification of patients in the supervised approach was due to the intrinsic nature of the supervised approach using the diagnostic label or because of the small sample size for building a classification model in the present study.

**Figure 9:**
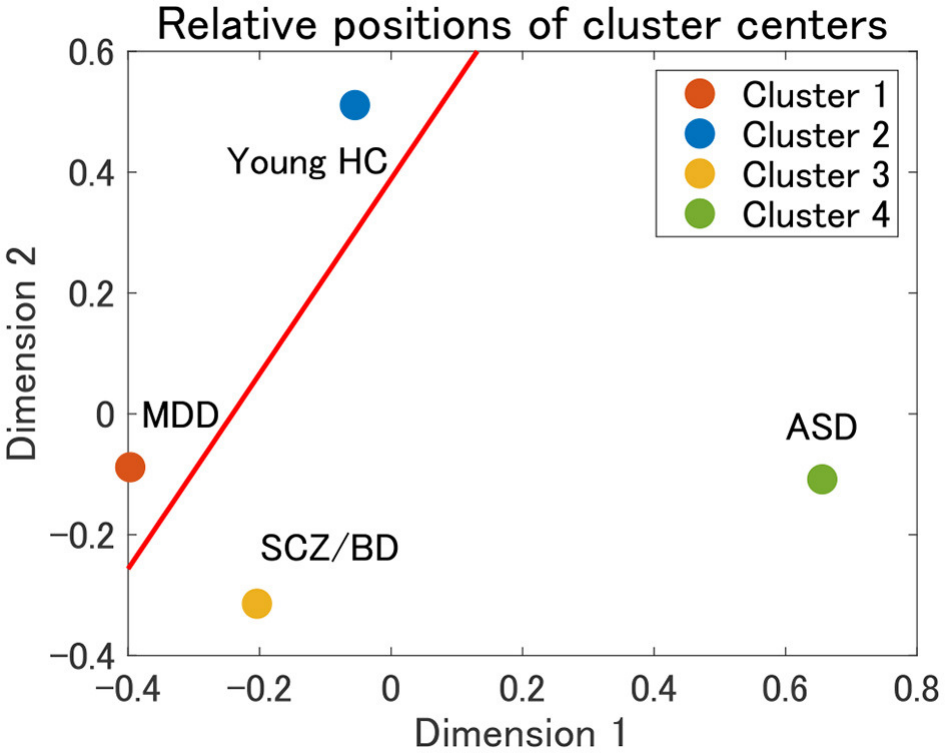
Relative positions of cluster centers in view 4 by means of multidimensional scaling (MDS). The red line denotes a possible axis for the continuum of clusters 1–3, which is a linear regression line for the centers of clusters 1–3.

Finally, the present cluster analysis provides a useful framework for the dimensional approach to psychiatric disorders. To the best of our knowledge, there have been few attempts to structurally elucidate the relationships among various psychiatric disorders (12). Thus, we consider one possible attempt to project cluster centers onto a two-dimensional plane using multidimensional scaling (MDS). MDS is a dimension-reduction method that preserves the distance between objects (58). In the present study, we use the Euclidean distance between the mean correlation matrices for cluster centers. The MDS results for clusters 1–4 in view 4 suggest that cluster 1 (MDD) and cluster 3 (SCZ/BD) are located nearby, whereas cluster 2 (young HC) and cluster 4 (ASD) are far apart (Figure 9). A closer look at the figure shows that young HC, MDD, and SCZ/BD are in the same continuum (red line), in which MDD is slightly closer to young HC than SCZ/BD. ASD is not located in this continuum, which suggests that it may comprise its own dimension. This interpretation of ASD is consistent with our finding that the depression scale (CES-D) of HC subjects is lower in cluster 4 than in clusters 2 and 3, implying less depressiveness for ASD.

### 4.4. Limitations

The first major limitation of the present study was the small sample size for psychiatric disorders, which lowered the statistical power for the characterization of the yielded clusters. The reason for the small sample size was that we focused on the specific site or scanner for the discovery data to alleviate the issue of site or scanner biases. Second, the main characterization of the yielded clusters was based on single diagnostic label information, due to the limited availability of other clinical information of the subjects. With the availability of more clinical data, it would be possible to characterize the clusters in a more comprehensive manner. Third, we did not remove the effects of confounding factors, such as age and sex. This was due to the intrinsic nature of the ROI-based multiple clustering method (not due to the FC-based method), which fitted a correlation matrix to the Wishart distribution. Note that the Wishart distribution required an input matrix to satisfy the positive-definite condition. This strict condition on the input matrix did not allow us to perform arithmetic operations for the matrix in an element-wise manner. Hence, the conventional GLM approach (57) to remove the confounding effect in an element-wise manner was not readily applicable to the present framework. For the same reason, it would not be straightforward to perform harmonization to remove the site or scanner bias, such as ComBat (59) and TS (57). It will be important for future work to overcome these difficulties for the ROI-based multiple clustering method.

## Data Availability Statement

Publicly available datasets were analyzed in this study. This data can be found at: https://bicr-resource.atr.jp/srpbsfc/.

## Ethics Statement

Ethical review and approval was not required for the study on human participants in accordance with the local legislation and institutional requirements. Written informed consent for participation was not required for this study in accordance with the national legislation and the institutional requirements.

## Author Contributions

TT, OY, YS, and JY contributed to conception and design of the study. TT wrote the first draft of the manuscript. All authors contributed to manuscript revision, read, and approved the submitted version.

## Conflict of Interest

The authors declare that the research was conducted in the absence of any commercial or financial relationships that could be construed as a potential conflict of interest.

## Publisher's Note

All claims expressed in this article are solely those of the authors and do not necessarily represent those of their affiliated organizations, or those of the publisher, the editors and the reviewers. Any product that may be evaluated in this article, or claim that may be made by its manufacturer, is not guaranteed or endorsed by the publisher.

## Footnotes

^1^*SRPBS Multidisorder Connectivity Dataset*. Available online at: https://bicrresource.atr.jp/srpbsfc/
