# Supplementary material for: Clustering of Multiple Psychiatric Disorders Using Functional Connectivity in the Data-Driven Brain Subnetwork
Source: Front Psychiatry. 2021 Aug 18;12:683280. doi: 10.3389/fpsyt.2021.683280 (PMC8416352; doi:10.3389/fpsyt.2021.683280)
Supplement: Supplementary file 1 [file Data_Sheet_1.PDF]

Supplementary Materials: Clustering of multiple psychiatric disorders using functional connectivity in the data-driven brain subnetwork

Tomoki Tokuda [*, 1, 4], Okito Yamashita [1, 3], Yuki Sakai[1], and Junichiro Yoshimoto [2, 1]

[1]Brain Information Communication Research Laboratory Group, Advanced Telecommunications Research Institute International, 2-2-2 Hikaridai, Seika-cho, Soraku-gun, Kyoto 619-0288, Japan
[2]Nara Institute of Science and Technology, 8916-5 Takayama, Ikoma, Nara 630-0192, Japan
[3]Center for Advanced Intelligence Project, RIKEN, Nihonbashi 1-chome Mitsui Building, 15th floor, 1-4-1 Nihonbashi, Chuo-ku, Tokyo 103-0027, Japan
[4]Okinawa Institute of Science and Technology Graduate University, 1919-1 Tancha, Okinawa 904-0495, Japan
[*]Corresponding author, t-tokuda@atr.jp

Table S1: Summary of classification of traveling subjects (TS) for view 4. Each subject undertook fMRI scanning three times (s1-3 in an arbitrary order) for the UTO scanner and the KYO-A scanner, respectively. Classification results (the digit in the table denotes cluster ID) are based on the model obtained by fitting the ROI-based multiple-clustering method to UTO data.

		UTO scanner			KYO-A scanner		
TS ID	Age	s1	s2	s3	s1	s2	s3
1	25	4	4	3	3	4	3
2	25	2	2	2	1	2	2
3	26	1	1	1	1	2	1
4	27	4	2	4	3	4	2
5	28	4	4	4	1	2	2
6	32	1	2	2	2	2	2
7	30	2	5	5	2	5	2
8	24	1	3	2	3	2	1
9	26	3	2	2	1	1	2

Table S2: The cluster-wise total number of subjects for classification of traveling subjects (TS)

Cluster ID	UTO scanner	KYO-A scanner
1	5	7
2	10	13
3	3	4
4	7	2
5	2	1

Table S3: Results of the elastic net classification for UTO data without regressing-out of age and sex (classification 1) and with regressing-out of age and sex (classification 2). Each cell denotes the number of subjects, which is specific for the psychiatric label (row) and the supervised classification (column).

Diagonosis Labels	Classification 1					Classification 2				
	HC	MDD	SCZ	BD	ASD	HC	MDD	SCZ	BD	ASD
HC	105	13	24	16	12	95	12	19	9	35
MDD	11	12	20	16	3	12	4	21	9	16
SCZ	5	9	7	10	4	4	6	8	6	11
BD	5	4	19	9	4	1	7	12	6	15
ASD	3	4	3	0	0	4	1	3	0	2

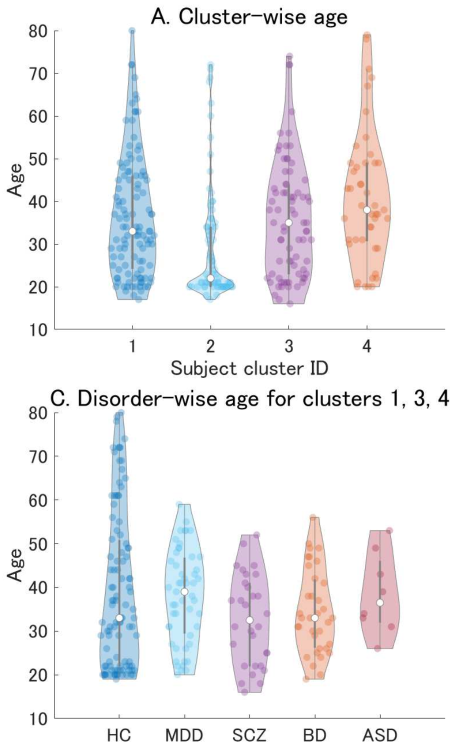

Figure S1: Age distribution of UTO subjects for view 4: Panel (**A**): Cluster-wise age distribution of subjects. Panel (**B**): Disorder-wise age distribution of subjects in clusters 1-4. Panel (**C**): Disorder-wise age distribution of subjects in clusters 1, 3, and 4. Panel (**D**): Disorder-wise age distribution of subjects in cluster 2.

c.1 vs. c.2

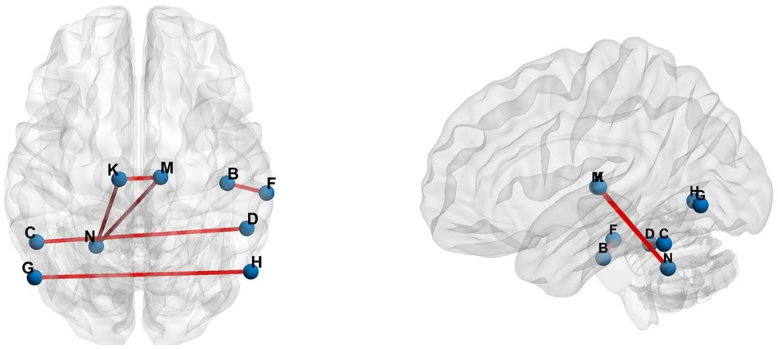

c.1 vs. c.3

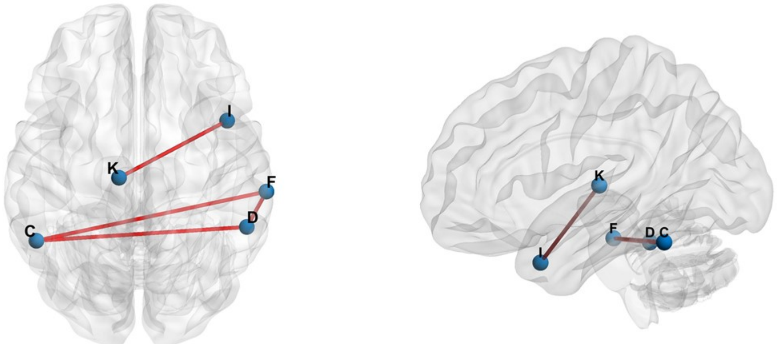

c.1 vs. c.4

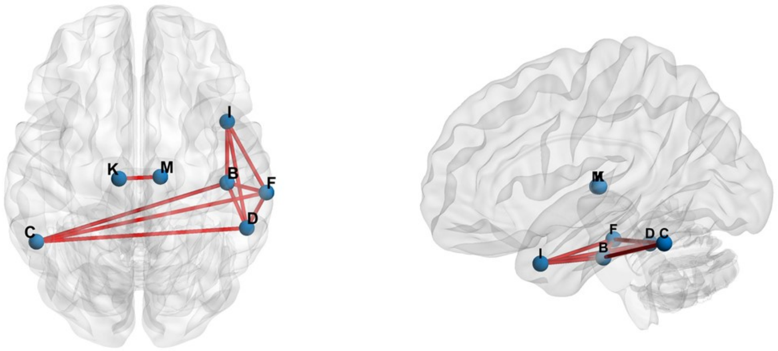

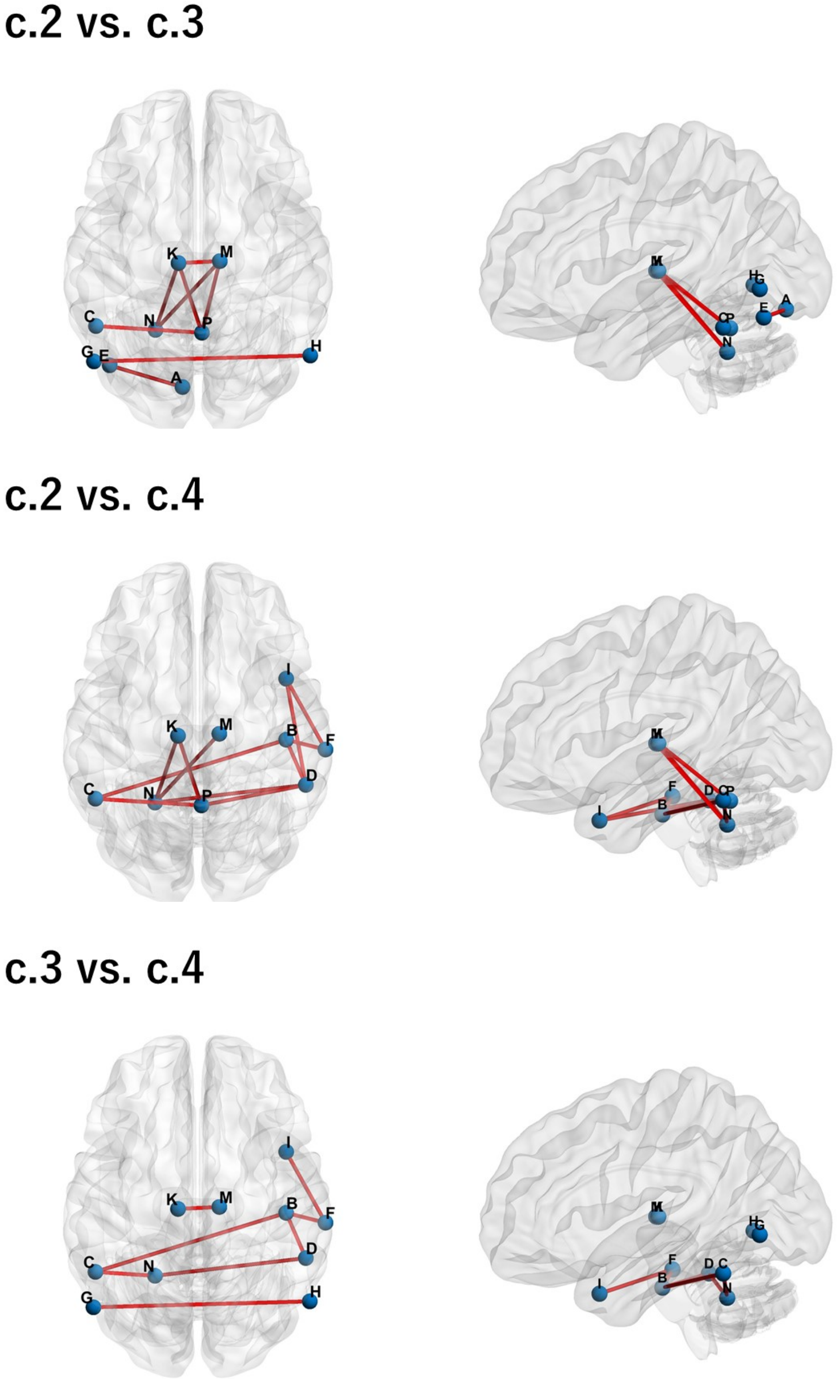

Figure S2: Visualization of the relevant connectivity of Figure 4 in the main manuscript. The alphabets denote the following ROIs. A (Left posterior intralingual sulcus); B (Right anterior occipito-temporal lateral sulcus); C (Left median occipito-temporal lateral sulcus); D (Right median occipito-temporal lateral sulcus); E (Left posterior occipito-temporal lateral sulcus); F (Right anterior inferior temporal sulcus); G (Left posterior inferior temporal sulcus); H (Right posterior inferior temporal sulcus): I (Right polar temporal sulcus); J (Left superior temporal sulcus); K (Left thalamus); L (Left pallidum); M (Right thalamus); N (Left cerebellum); P (Vermis).

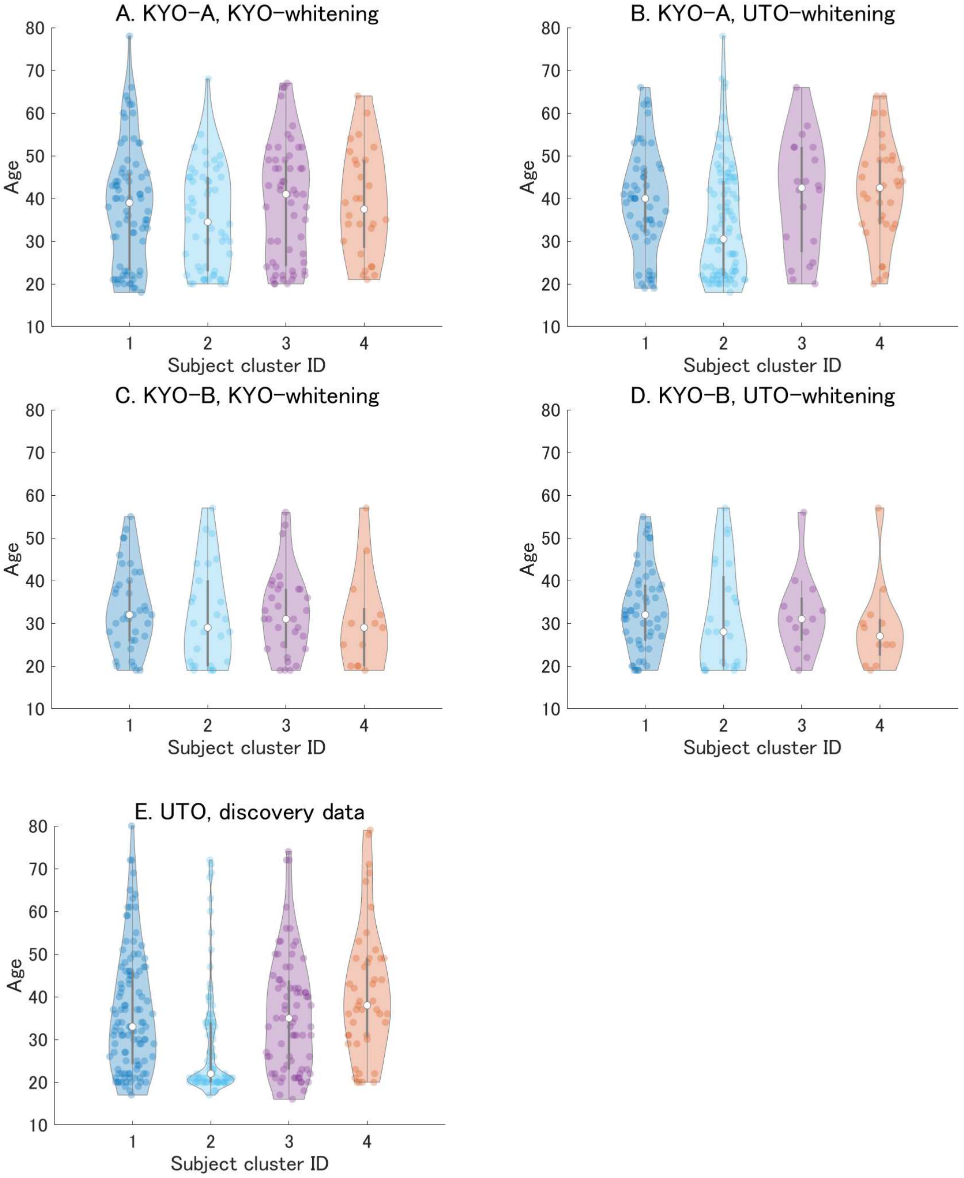

Figure S3: Age distribution of KYO subjects that are classified by the yielded model from UTO data. Panel (**A**), (**B**): Age distribution for KYO-A data with KYO-whitening and with UTO-whitening, respectively. Panel (**C**), (**D**): Similar graphs for KYO-B data. Panel (**E**): This is a copy of Figure S1(**A**) for the purpose of comparison.

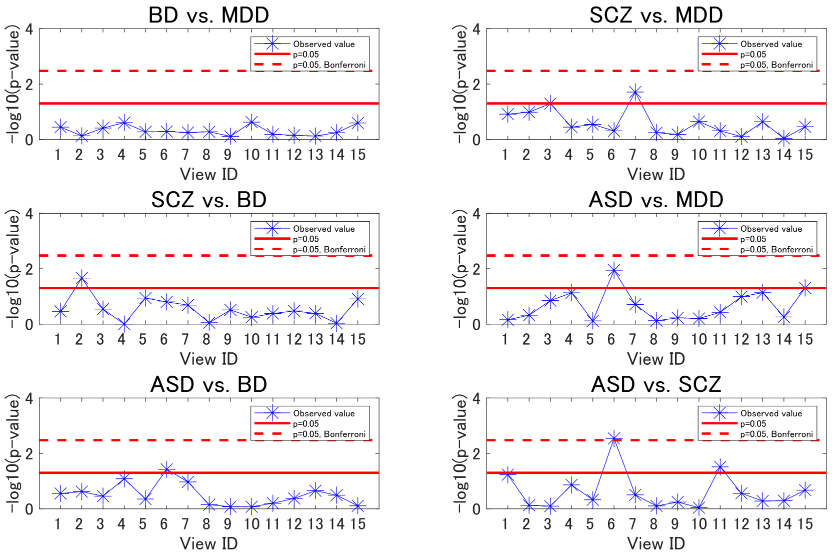

Figure S4: Association between cluster labels and pairs of psychiatric disorders for UTO-dataset. The χ^2 test is performed to test the association between cluster labels and a particular pair of psychiatric disorders displayed on the top of each panel. In this analysis, we focused on the clusters with size greater than 9 (using all subjects).
